# Editorial: Interplay Between Ion Channels, the Nervous System, and Embryonic Development

**DOI:** 10.3389/fnmol.2021.618815

**Published:** 2021-03-24

**Authors:** Michael Levin, Angeles B. Ribera

**Affiliations:** ^1^Department of Biology and Allen Discovery Center at Tufts University, Medford, MA, United States; ^2^Department of Physiology and Biophysics, University of Colorado School of Medicine, Denver, CO, United States

**Keywords:** ion channels, development, embryo, nervous system, bioelectricity

Ion channels play fundamental second-to-second roles in maintaining appropriate ion gradients and membrane potentials for all cells, enabling rapid signaling in excitable cells and standing patterns of voltage in non-excitable tissues. Recent work has revealed that ion channels play critical roles in development of excitable and non-excitable cells and organs, neural degeneration, disease and tissue remodeling/cellular regeneration (Bates, 2015). Examples of ion channels with associated channelopathies that have revealed such novel roles include Kv3.1, Kir2.1, Kir7.1, and GIRK2 (Belus et al., 2018). Elucidation of these non-conventional roles of ion channels will provide entree' into novel mechanisms that underlie development, degeneration and/or regeneration of excitable and non-excitable cells (Levin and Martyniuk, 2018). Moreover, as ion channels serve as potential drug targets, elucidation of their non-traditional roles will allow for innovative new therapeutic approaches targeting bioelectric state (Churchill et al., 2018), as has already been shown for regenerative applications (Tseng et al., 2010) and cancer (Arcangeli et al., 2012).

This Research Topic focuses on non-conventional roles played by ion channels in development, disease, and repair of excitable and non-excitable tissues. Here, we briefly summarize the areas covered by each article in this collection. Together, the articles cover a wide spectrum of issues in the field.

The disease conditions that arise from mutations in ion channels are known as channelopathies. Tyagi et al. review the diverse range of channelopathy symptoms that arise from the numerous mutations that have been discovered in the human gene *CACNA1A* that encodes the calcium channel Cav2.1. An important major point of their review is the need to determine not only the direct effect of the mutation on channel function as well as the effect of the mutant channel in a physiological context. When studied in the “reduced” context of a heterologous expression system, the direct biophysical effect of a mutation on channel function is most easily discerned. However, it is often difficult to extrapolate the ultimate consequence of the mutant channel when it functions in a native context. In this regard, they highlight the advantages of the zebrafish as a model system for study of the mechanistic basis of the effects of calcium channelopathies. The zebrafish model has previously advanced a mechanistic understanding of how mutations in the human *SCN1A* sodium channel have the potential to alter cellular and network excitability and lead to Dravet syndrome (Hortopan et al., 2010). These considerations need to be kept in mind for interpretation and inferences regarding implications for therapeutic approaches. Moreover, the zebrafish model has shown that ion channel effects can be exerted non-cell autonomously as well as cell autonomously (Pineda et al., 2006; Wright and Ribera, 2010). The paper forms a nice complement to past studies of developmental roles of channels in zebrafish morphogenesis and its disorders (Perathoner et al., 2014; Silic and Zhang, 2018; Lanni et al., 2019).

Non-excitable as well as excitable cells express ion channels, often during early stages of differentiation. Ozekin et al. focus on the ion channel Kir2.1 and its role in bone development with a focus on craniofacial morphogenesis. Interestingly, this ion channel has also been implicated in other developmental processes, e.g., *Drosophila* wing development (Dahal et al., 2012, 2017; George et al., 2019). Importantly, several teratogens target ion channels that play development roles (Hernandez-Diaz and Levin, 2014). The recent vaping surge highlights the importance of understanding the effects of nicotine on ion channels that play developmental roles. The studies reviewed highlight the importance of the concept of “developmental bioelectricity” in the context of the embryo.

Goyal et al. review studies of the ontogeny of ion currents in developing nervous systems. They highlight roles of ion channels in neural tube formation, and the molecular mechanisms downstream of their function that guide cell proliferation and differentiation (Spitzer et al., 2013). The regulation of morphogenetic and cellular processes by electrical activity is shown to be critical for the development of the vertebrate nervous system.

Pai et al. study the role of bioelectric prepatterns in establishment of the correct size and shape of the frog embryo brain. Prior work has shown that mutations and exposure to teratogens can cause developmental malformations via disruption of the sharp voltage differences between compartments of the endogenous bioelectric prepattern that sets the size and shape of the brain (Pai et al., 2015a,b, 2017, 2018). Here, they show that up-regulation of context-sensitive channels, such as HCN2 (identified by a computational strategy), sharpens voltage boundaries and rescues the normal bioelectric prepattern, which in turn restores gene expression, brain morphogenesis, and cognitive function despite the presence of strong teratogens. They use a computational model to predict, and validate, repair via HCN2 expression induced in tissues far away from the brain (such as in the ventral gut region), or via systemic exposure to drugs already approved for human use.

Alicea reviews the cell and molecular data on the origin of the invariant structure of the *C. elegans* embryo. Studying the connectome of the emerging organism (Barabasi and Barabasi, 2020), they propose a model of synaptic connectivity that reveals design principles of electrical networks, which help understand the life and function of this important model system. Characterizing these earliest events of the cooperation of the genetic and biophysical layers is critical for both understanding the ontogeny of bioelectric gradients and for fully appreciating the evolutionary dynamics that can take advantage of the interplay between these two layers of morphogenetic control.

Together, these studies reveal the richness of the emerging roles of ion channel activity in health and disease. These studies contribute to a field with many implications for basic knowledge about the processing of information by biophysical mechanisms in living tissue, driving biomedical applications based on modulating ionic communication during health and disease.

## Author Contributions

ML and AR wrote the manuscript and approved the final version for publication. Both authors contributed to the article and approved the submitted version.

## Conflict of Interest

The authors declare that the research was conducted in the absence of any commercial or financial relationships that could be construed as a potential conflict of interest.
